# The impact of the EVLP on the lung microbiome and its inflammatory reaction

**DOI:** 10.3389/ti.2024.12979

**Published:** 2024-11-11

**Authors:** Leandro Grando, Marc Boada, Rosa Faner, Susana Gómez-Ollés, Victoria Ruiz, Marc Bohils, Joaquim Albiol, Ramses Marrero, Laia Rosell, Ivan Salinas, Daniel Ruiz, Ángel Ruiz, Camino Rodríguez-Villar, Anna Ureña, David Paredes-Zapata, Ángela Guirao, Gerard Sánchez-Etayo, Laureano Molins, Néstor Quiroga, Aroa Gómez-Brey, Xavier Michavila, Alberto Sandiumenge, Àlvar Agustí, Ricard Ramos, Irene Bello

**Affiliations:** ^1^ Department of Thoracic Surgery, Respiratory Institute, Hospital Clínic, Barcelona, Spain; ^2^ Universitat de Barcelona, Catedra Salut Respiratoria, Barcelona, Catalunya, Spain; ^3^ CIBER Enfermedades Respiratorias (CIBERES), Madrid, Spain; ^4^ Institut d’Investigacions BIomediques August Pi i Sunyer (IDIBAPS), Barcelona, Spain; ^5^ Universitat de Barcelona, Biomedicine Department, Barcelona, Catalunya, Spain; ^6^ Department of Pneumology, Vall d’Hebron Institut de Recerca, Barcelona, Spain; ^7^ Department of Donor and Transplant Coordination, Hospital Clínic, Barcelona, Spain; ^8^ Department of Anaesthesia and Perioperative Care, Hospital Clínic of Barcelona, Barcelona, Spain; ^9^ Surgical Area, Hospital Clínic, Barcelona, Spain; ^10^ Department of Donor and Transplant Coordination, Vall d’Hebron University Hospital, Barcelona, Spain; ^11^ Department of Pneumology, Respiratory Institute, Hospital Clínic, Barcelona, Spain

**Keywords:** *ex vivo* lung perfusion, EVLP, lung microbiome, primary graft dysfunction, lung donor, protocol study, inflammatory response

## Abstract

The pulmonary microbiome has emerged as a significant factor in respiratory health and diseases. Despite the sterile conditions maintained during *ex vivo* lung perfusion (EVLP), the use of antibiotics in the perfuse liquid can lead to dynamic changes in the lung microbiome. Here, we present the design of a study that aims to investigate the hypothesis that EVLP alters the lung microbiome and induces tissue inflammation. This pilot, prospective, controlled study will be conducted in two Spanish donor centers and will include seven organ donors after brain death or after controlled cardiac death. After standardized retrieval, the left lung will be preserved in cold storage and the right lung will be perfused with EVLP. Samples from bronchoalveolar lavage, perfusion and preservation solutions, and lung biopsies will be collected from both lungs and changes in lung microbiome and inflammatory response will be compared.

## Introduction

Lung transplantation (LTx) is the ultimate treatment option for patients suffering from several end-stage pulmonary diseases. Despite efforts to optimize results, primary graft dysfunction (PGD) is one of the most serious and common complications of LTx [1]. PGD is an inflammatory pulmonary response that occurs due to ischemic-reperfusion damage. It is associated with both early mortality [2] and long-term outcomes, including chronic lung allograft dysfunction and late mortality.

The lungs harbor a diverse community of microbes, collectively known as the pulmonary microbiome [3]. This microbial ecosystem, intricately intertwined with the local immune landscape, exerts a profound influence on pulmonary homeostasis and inflammatory responses [4]. Alterations of the microbial composition and diversity of the pulmonary microbiome (dysbiosis) have been associated with aberrant immune activation and sustained inflammation, contributing to the pathogenesis of acute and chronic respiratory disorders [5].

*Ex vivo* lung perfusion (EVLP) [6] offers a platform for pre-transplant organ assessment, optimization, and repair. It provides the opportunity to evaluate marginal donor lungs, mitigate ischemia-reperfusion injury, and even rehabilitate injured or rejected lungs deemed unsuitable for transplantation under static conditions.

## Methods

### Hypothesis

We hypothesize that lung perfusion with Steen’s solution, blood, and antibiotics, with the addition of temperature changes, can alter the lung microbiome and improve microbiome homeostasis. We aim to investigate this and analyze if these changes affect the inflammatory environment.

### Objectives

The primary objective of this study is to understand the impact of EVLP on the lung microbiome and tissue inflammation as compared with cold ischemia.

The objectives are:

• To describe the changes in the lung microbiome with cold ischemia
• To analyze the changes in lung microbiome with ELVP
• To discover if EVLP or cold storage improve the microbiome environment
• To compare the changes in the lung microbiome between cold ischemia preservation and EVLP
• To analyze the changes in pulmonary inflammatory parameters with cold ischemia due to lung microbiome changes
• To investigate the changes in pulmonary inflammatory parameters with EVLP due to lung microbiome changes
• To contrast the changes in inflammatory parameters between cold storage and EVLP due to lung microbiome changes

### Study Design and Ethics

This pilot, prospective, and controlled study will be conducted in two Spanish donor centers. The study will compare the microbiome of the right lung before and after EVLP to understand the changes that occur and the microbiome of the left lung before and after cold storage. The final microbiome of both lungs will be compared. The current clinical practice actually is cold storage; it is for this reason that we will compare the EVLP changes with the cold storage changes.

The study protocol complies with the 1975 Declaration of Helsinki and has been approved by the Human Research Committees [HCB/2022/0024 and PR(AG)106/2019]. Informed consent to participate in the study was included in the donation consent form. The protocol was registered at ClinicalTrials.gov, NCT06250517.

### Study Population

We will include seven organ donors after death determined by neurologic criteria (DBD) or by circulatory criteria (DCD) Maastricht type III. The type of death will be certified in accordance with Spanish legislation.

Lungs included in the study will be older than 18yo and discarded for transplantation by all Spanish and Europe lung transplant programs; the cause of rejection will be recorded. The exclusion criterion is the presence of unilateral radiological alteration or unilateral pneumonia.

### Lung Preparation Procedure Prior to the Preservation Protocol

Before bronchoscopy, in the operation room, a sample of sterile saline solution will be taken through the fiberoptic bronchoscope as a negative control of microorganisms in the bronchoscope (BAL_Tx_). A bronchoalveolar lavage (BAL) of 40 mL will be collected from the left main bronchus (BALL_T0_). The lungs will be perfused with 60 mL/kg of Perfadex Plus (*XVIVO Perfusion AB, Sweden*) and they will be retrieved following clinical practice. The lungs will be split through the left bronchus using a staple.

**FIGURE 1:**
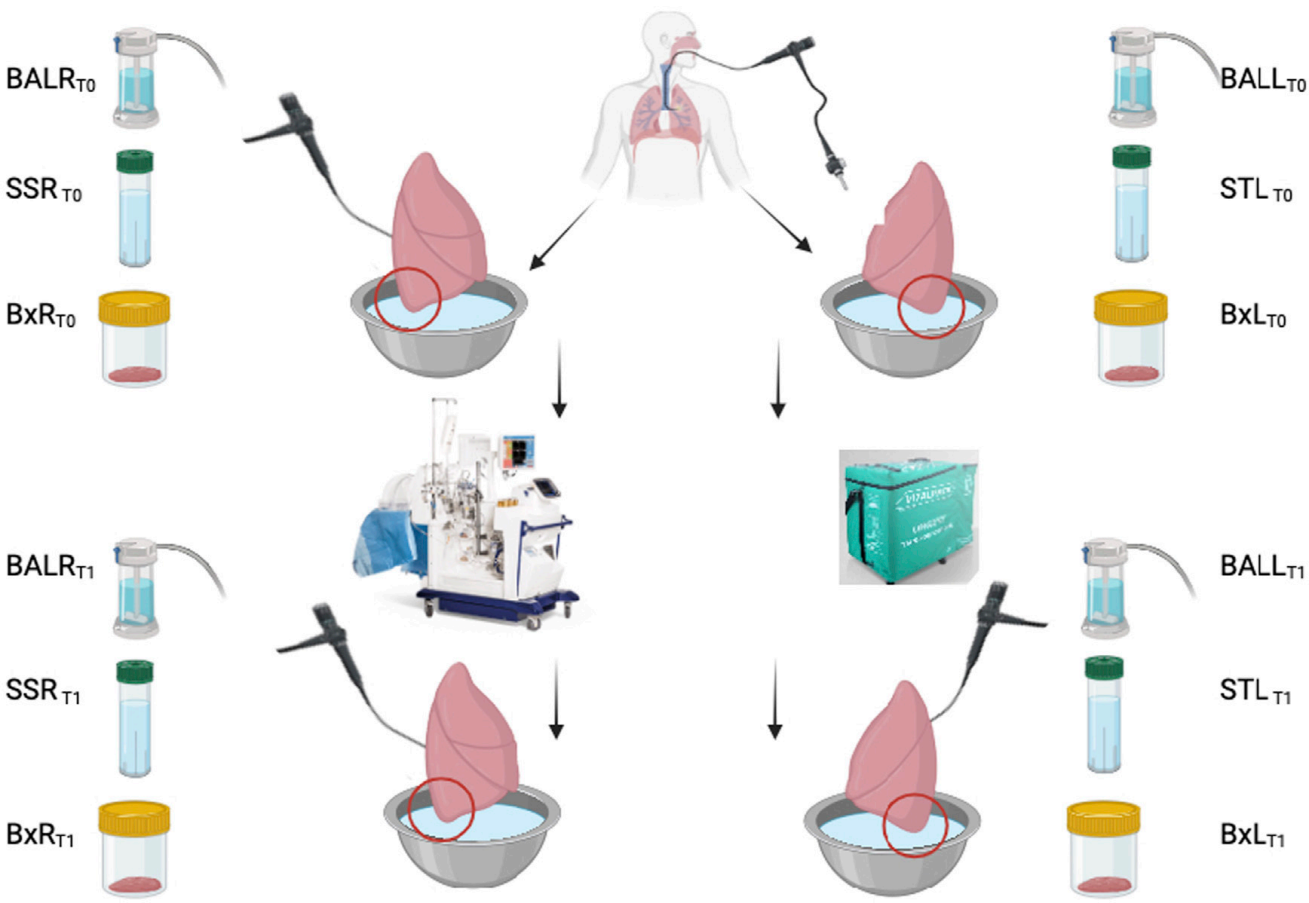
Protocol scheme.

The left lung will be preserved in cold storage and the right lung will be perfused with EVLP (Figure 1).

### Cold Storage Protocol and Sample Obtention (Control Group)

The left lung will be preserved with 2L of Perfadex Plus^®^ (*XVIVO Perfusion AB, Sweden*) in cold storage at 4°C in a Vitalpack^®^ container for 3 h.

Before the cold storage of the left lung, a 50 mL sample of the transport solution (STL _T0_) will be collected. A 3 cm lung biopsy from the lower lobe will be taken using a staple (BxL_T0_) and preserved with RNAlater.

After 3 h of cold storage, the storage procedure will be considered completed. The left main bronchus will be opened, a 50 mL BAL will be collected (BALL_T1_), and a lower lobe lung biopsy (BxL_T1_) will be collected and preserved with RNAlater. A sample of 50 mL of the transport solution (STL_T1_) will be recovered.

### EVLP Protocol and Sample Obtention (Intervention Group)

The right lung will be perfused for 3 h with LS™(*XVIVO Perfusion AB, Sweden*).

Before EVLP, 50 mL of BAL from the main right bronchus (BALR_T0_) and 50 mL of perfusion liquid (SSR _T0_) will be collected and one lung biopsy of 3 cm using a staple from the lower lobe will be taken (BxR_T0_) and preserved with RNAlater.

The EVLP will be initiated 3 h after Lund’s protocol [7]. The perfusion liquid will be 2L of Steen Solution^®^ (*XVIVO Perfusion AB, Sweden*), 1 red blood cell pack of donor’s isogroup, and 1g of meropenem.

After 3 h of EVLP, the protocol will be finalized. A BAL of 50 mL from the right main bronchus (BALR_T1_) and a sample of 50 mL of the perfusion solution (SSR_T1_) will be collected; one lung biopsy of the lower lobe will be taken (BxR_T1_) and preserved with RNAlater.

### Data Collection and Sample Analysis

The data collection will be from the sample and the donor characteristics (Table 1). Table 1 lists what data will be collected in both groups.

**TABLE 1:** Data collection.

Sample data	Donor data
• Microbiome before EVLP in right graft • Microbiome before cold storage in left graft • Microbiome after EVLP in right graft • Microbiome after cold storage in left graft • RNA before EVLP in right graft • RNA before cold storage in left graft • RNA after EVLP in right graft • RNA after cold storage in left graft • Cytokines after EVLP in right graft • Cytokines after cold storage in left graft	• Age • Gender • Cause of death • Length of stay in ICU • Days of intubation • Intubation site • Cause for rejection of lung donation • Respiratory cultures • Antibiotic therapy • History of bronchoaspiration • PaFi before the retrieval (FiO2 1 and PEEP 5)

#### Microbiome Analysis

For the extraction of genomic DNA, the protocol that was previously used in the MetaHIT project will be applied [8]. For the analysis of microbial diversity, the V4 hypervariable region of the 16S rRNA gene will be amplified from bacterial DNA by PCR using the universal primers described in Pozuelo et al. [9]. Amplicons will be sequenced using Illumina (MiSeq) technology.

To investigate the microbial composition of each sample, raw sequence reads will be demultiplexed using the idem tool. The resulting single-end reads will be processed to obtain an amplicon sequence variant (ASV) table, to which taxonomy will be assigned by using SILVA 16s rRNA database (v. 132). Alpha diversity metrics, including Observed and Shannon diversity indices, and beta diversity metrics such as the Bray Curtis and Aitchison distance indices are going to be calculated. The Aitchison distance between samples will be calculated using the CodaSeq (v.0.99.6) and zComposition (v. 1.3.4) R packages. Data is going to be normalized by transforming the raw counts to centered log-ratios (clr).

#### Analysis of Genetic Expression

RNA extraction will be performed using the commercial RNeasy Mini Kit (QIAGEN). Gene expression analysis will be performed by quantitative PCR (qPCR) using the predesigned TransplantRejection panel from SignArray (AnyGenes^®^, Paris, France), which includes 84 genes that have been described to be related to the immune response in transplant rejection. The genes included in the panel are CX3CR1, ICAM1, ITGA2, ITGAE, ITGAM, PECAM1, THBS1, THBS2, VCAM1, COL1A2, CCR5, CCR7, CD40, CD40LG, CD80, CD86, CTLA4, CXCR3, STAT4, TGFB1, CD44, CTGF, MMP1, MMP2, MMP7, MMP9, BMP7, CCL11, CCL2, CCL3, CCL4, CCL5, CSF2, CXCL10, IFNG, IL10, IL12A, IL13, IL16, IL1B, IL2, IL2RA, IL3, IL32, IL4, IL5, IL6, IL8, TNF, TGFB2, TGFB3, TIMP1, VEGFA, MS4A1, CXCL11, CXCL9, CXCR4, ADAM17, C3, CASP1, CASP3, CASP8, CCR2, CCR3, CD14, CD28, CD8A, FAS, FASLG, FCGR1A, GZMA, GZMB, NFKB1, NOS2, PRF1, PSMB9, STAT1, STAT6, TAP1, TLR3, TLR4, TLR9, TNFAIP3, and TNFSF10.

#### Cytokine Analysis

The determination of cytokines in the perfusion fluid will be carried out using immunoassays based on Luminex™ xMAP™ technology (multi-analyte profiling). We will use panels designed specifically for the gene products of interest. Cytokine levels are measured using an immunoassay based on Luminex™ xMAP™ technology, which allows multiparametric analysis of the different cytokines. The cytokines analyzed in the panel designed for this purpose are IFN gamma, IL-1 beta, IL-6, IL-8 (CXCL8), IL-18, IP-10 (CXCL10), MCP-1 (CCL2), MIP- 1 alpha (CCL3), TNF alpha, and VEGF-D (Custom Procartaplex Multiplex Panel, Invitrogen, ThermoFisher Scientific, MA, United States). The immunological analysis will be carried out using the Invitrogen ProcartaPlex Analyst 1.0 software, supplied with the reagents. The trials will be carried out blind to the clinical evolution of the recipients.

### Statistical Analysis

Since this is a pilot study, no formal sample size calculation was undertaken. Categorical variables are going to be expressed as number of cases and percentage and will be compared using chi-square test or Fisher’s exact test. After checking for normality using the Shapiro-Wilk test, continuous variables will be expressed as median and interquartile range (IQR) or mean ± standard deviation as appropriate. To compare variables across groups, Mann-Whitney U test or Student’s t-test are going to be used. To compare within the same condition before and after preservation time points, Wilcoxon signed rank test or paired *t*-test will be carried out. The inflammatory response will be reported by analyzing the quantitative PCR of the genetic expression of 84 genes related to immune response in lung biopsy and the concentration of cytokines in perfusion and preservation solutions before and after EVLP and cold storage.

## Discussion

PGD is the most common cause of 30-day mortality after lung transplantation and the second most common after the first year [8]. It is a complex syndrome characterized by severe hypoxemia and pulmonary edema occurring within the initial 72 h post-transplantation [9]. Understanding the underlying pathophysiological mechanisms, including ischemia-reperfusion injury, innate immune activation, endothelial dysfunction, and inflammatory response [10], is essential for developing targeted therapeutic strategies that can mitigate PGD-associated complications. Furthermore, elucidating predictive biomarkers and risk factors for PGD may enable risk stratification and preventive interventions, thereby potentially improving graft and patient outcomes.

The pulmonary microbiome has emerged as a significant factor in respiratory health and disease [11]. Its role extends beyond local immune modulation within the lungs to potentially impacting systemic health.

Sharma et al. [4] observed that some microbiome signatures of distal airways promote an inflammatory reaction and contribute to the development of BOS. Prakash et al. [12] found in a murine model that inflammation generated by left lung ischemia-reperfusion dissipated after antibiotic treatment due to the depletion of intestinal microbiota. Also, alveolar macrophages can be modulated by the microbiota. Bernasconi et al. [13] showed that the bacterial dysbiosis between proinflammatory and anti-inflammatory communities were linked to inflammatory and remodeling processes. Furthermore, recent studies from McGinnins et al. [14] found a relationship between the presence of dysbiosis with an alteration *Prevotella/Streptococcus* ratio and severe PGD after lung transplantation.

EVLP is a form of isolated lung perfusion in normothermic conditions and can be achieved with a pump-driven perfusion machine that recirculates a preservation solution through the vasculature of the lung in addition to mechanical ventilation. This technology was initially developed to evaluate pulmonary donor grafts prior to transplantation and was successfully introduced in clinical practice. In addition, there is also growing evidence that EVLP could serve as a potential dynamic preservation strategy; it could also serve as a platform to actively resuscitate or recondition lungs while metabolically active. Currently, two different main protocols exist: the Toronto and Lund protocols. The question of whether the Lund or Toronto protocol for *ex vivo* lung perfusion (EVLP) is superior is complex and multifaceted, as both protocols have shown significant advantages and benefits in different aspects of lung transplantation.

The Toronto protocol, which has been widely studied and implemented, emphasizes the use of normothermic EVLP to assess and recondition marginal donor lungs, thereby increasing the pool of suitable donor organs and improving transplant outcomes. This protocol has demonstrated encouraging short- and medium-term outcomes, with no significant difference in long-term mortality, retransplantation rates, or chronic lung allograft dysfunction (CLAD) when compared to conventionally preserved lungs [15].

On the other hand, the Lund protocol has also shown promise, particularly in its ability to achieve stable multiday preservation of donor lungs through advanced technical modifications such as perfusate enrichment, low-flow, pulsatile, subnormothermic perfusion, and personalized ventilation strategies. These innovations could potentially lead to even better outcomes by allowing for more sophisticated treatment modalities during EVLP. Furthermore, the development of new perfusates, such as the DMEM-based nutrient-rich solution, has shown potential in enhancing cell viability and reducing apoptosis, which could be integrated into either protocol to further improve donor lung quality and quantity [16]. Overall, while both protocols have their strengths, the choice between them may depend on specific clinical scenarios and the availability of resources. One of the major differences between both protocols is the use of red blood cell packets. Current studies on the use of red blood cell packets in *ex vivo* lung perfusion (EVLP) highlight both their potential benefits. Red blood cells (RBCs) are traditionally used as oxygen carriers in EVLP due to their ability to support cellular metabolism during normothermic perfusion. We chose the Lund protocol with RBCs because this is our current EVLP protocol, and it will be our protocol chosen for long-term EVLP.

The lung inflammatory micro ambient can be influenced by several factors, including the type of donor, either DBD or cDCD, or the use of EVLP. Baciu [17] demonstrated that different donor-specific gene signatures depend on the type of donor and the use of EVLP. They found that lungs from donors who died from brain death (DBD) showed higher levels of inflammatory cytokines and pathways, indicating a strong inflammatory response in these lungs. In contrast, lungs from cDCD donors exhibited gene signatures related to cell death, apoptosis, and necrosis, suggesting these lungs are more prone to cell damage. They showed that EVLP could modify the transcriptomics. In DBD lungs, the tumor necrosis factor receptor-1/2 signaling pathways and macrophage migration inhibitory factor-associated pathways were activated. The study also identified a set of genes that can differentiate between DBD lungs treated with EVLP and those that were not. In our protocol, we will analyze the inflammatory parameters and different activated genes. To understand the influence of the microbiome we will compare them with the known genes.

EVLP presents a unique opportunity to explore the interactions between the pulmonary microbiome and graft viability. Despite the sterile conditions maintained during EVLP, the perfusion with antibiotics and Steen solution and the changes in temperature may cause dynamic changes in the lung microbiome [18]. Understanding the impact of EVLP on the pulmonary microbiome is essential to optimize transplant protocols and improve patient outcomes. Elucidating the complex interplay between *ex vivo* lung perfusion and the pulmonary microbiome may uncover novel strategies to enhance graft viability, prevent post-transplant complications, and ultimately improve the success rates of lung transplantation.

In summary, this will be the first study designed to analyze the impact of EVLP on the lung microbiome and the local inflammatory response. Understanding the composition, diversity, and functional interactions of the pulmonary microbiome in lung transplants holds promise for personalized respiratory medicine.
